# Retained Lippes Loop Intrauterine Device (IUD) in a Woman With Post-menopausal Bleeding and Chronic Pelvic Pain

**DOI:** 10.7759/cureus.106681

**Published:** 2026-04-08

**Authors:** Leila Neisani Samani, Elham Neisani Samani

**Affiliations:** 1 Department of Midwifery, Iran University of Medical Sciences, Tehran, IRN; 2 Department of Obstetrics and Gynaecology, Garden City Hospital, Garden City, USA

**Keywords:** intrauterine device, lippes loop, pelvic pain, post-menopausal bleeding, retained iud

## Abstract

Forgotten intrauterine devices (IUDs), particularly those retained for decades, can lead to complications in postmenopausal women, including bleeding and chronic pelvic pain. The Lippes Loop IUD, widely used from the 1960s to the 1980s, is now rarely encountered.

We present the case of a 68-year-old woman with a forgotten Lippes Loop IUD who presented with chronic pelvic pain and postmenopausal bleeding. The patient was uncertain whether her IUD had been spontaneously expelled or removed. During a laparoscopic hysterectomy performed for postmenopausal bleeding, an intra-pelvic Lippes Loop IUD was discovered embedded in the cul-de-sac and successfully removed. The patient tolerated the procedure well, and her postoperative period was uneventful. Her symptoms resolved completely at follow-up.

Removal of a retained IUD is highly recommended, particularly in postmenopausal women. Patients with an IUD presenting with postmenopausal bleeding and pelvic pain should be closely evaluated. We emphasize the importance of adequate counseling, regular follow-up for IUD users, and timely removal once the device has expired or is no longer indicated."

## Introduction

Intrauterine devices (IUDs) are one of the most effective, long-acting, and reversible methods of contraception, currently utilized by approximately 14.3% of contraceptive users worldwide [1,2]. Among the various IUD types, the Lippes Loop holds historical significance as a first-generation, non-medicated device made of polyethylene. Introduced by Dr. Jack Lippes in 1962, it was widely used from the 1960s through the 1980s before being discontinued [3,4]. Consequently, encountering a Lippes Loop in contemporary clinical practice is exceptionally rare and typically indicates prolonged, often "forgotten," retention. While generally safe, IUD use is associated with potential complications, including abnormal bleeding, infection, expulsion, and uterine perforation. Uterine perforation is an uncommon complication, with an estimated incidence of 1 in 1,000 insertions. In approximately 80% of perforation cases, the IUD is found partially or completely within the peritoneal cavity [3]. When an IUD is retained for many years, particularly after menopause, it may lead to chronic pelvic pain, bleeding, or diagnostic uncertainty. Management of a perforated or migrated IUD typically involves surgical removal, often via laparoscopy or laparotomy [3,4]. Here, we present a rare case of a forgotten Lippes Loop IUD discovered in the pelvic cavity of a 68-year-old postmenopausal woman during laparoscopic surgery. This case highlights the diagnostic challenges, clinical implications, and importance of timely management for long-retained intrauterine devices.

## Case presentation

A 68-year-old, gravida 5 para 5, postmenopausal woman presented for chronic pelvic pain for the last 2 years. She described her pain as constant, pressure-like, rated 5/10, and radiating to her back. She also reported vaginal spotting over the past three weeks. She denied any abnormal vaginal discharge, itching, or burning. Her last menstrual period was at 51 years old. Her past medical history was notable for hypertension, which was well-controlled on nifedipine 10 mg daily. The patient also reported anxiety on Zoloft 50 mg daily, but no depression. Surgical history was notable for three cesarean sections, an appendectomy, and a recent hysteroscopy with benign endometrial polyp removal. The patient reported having an IUD inserted for contraception during her reproductive years back home; however, she was uncertain whether it had been spontaneously expelled or removed. She could not recall the name of the IUD or the exact date of insertion. 

On physical examination, the patient was alert and oriented to time, person, and place. On physical examination, she was hemodynamically stable, with a soft, non-tender abdomen and no palpable masses. Gynecologic exam revealed a multiparous cervix, no lesions or bleeding, and a normal-sized uterus with no adnexal masses. On speculum examination, no visualized IUD string was visible. Laboratory values, including urinalysis, CBC, and blood glucose, were normal.

Transvaginal ultrasound showed a heterogeneous uterus measuring 65 mm x 25 mm x 35 mm. The endometrial stripe was 5 mm thick. We recommended an abdominal pelvic CT scan with and without contrast; however, the patient declined due to anxiety. Given her postmenopausal bleeding and prior benign endometrial polyp, she was counseled regarding diagnostic and therapeutic options and elected to proceed with a laparoscopic hysterectomy. After risks and benefits were discussed, the patient agreed to proceed with the procedure. 

**Figure 1:**
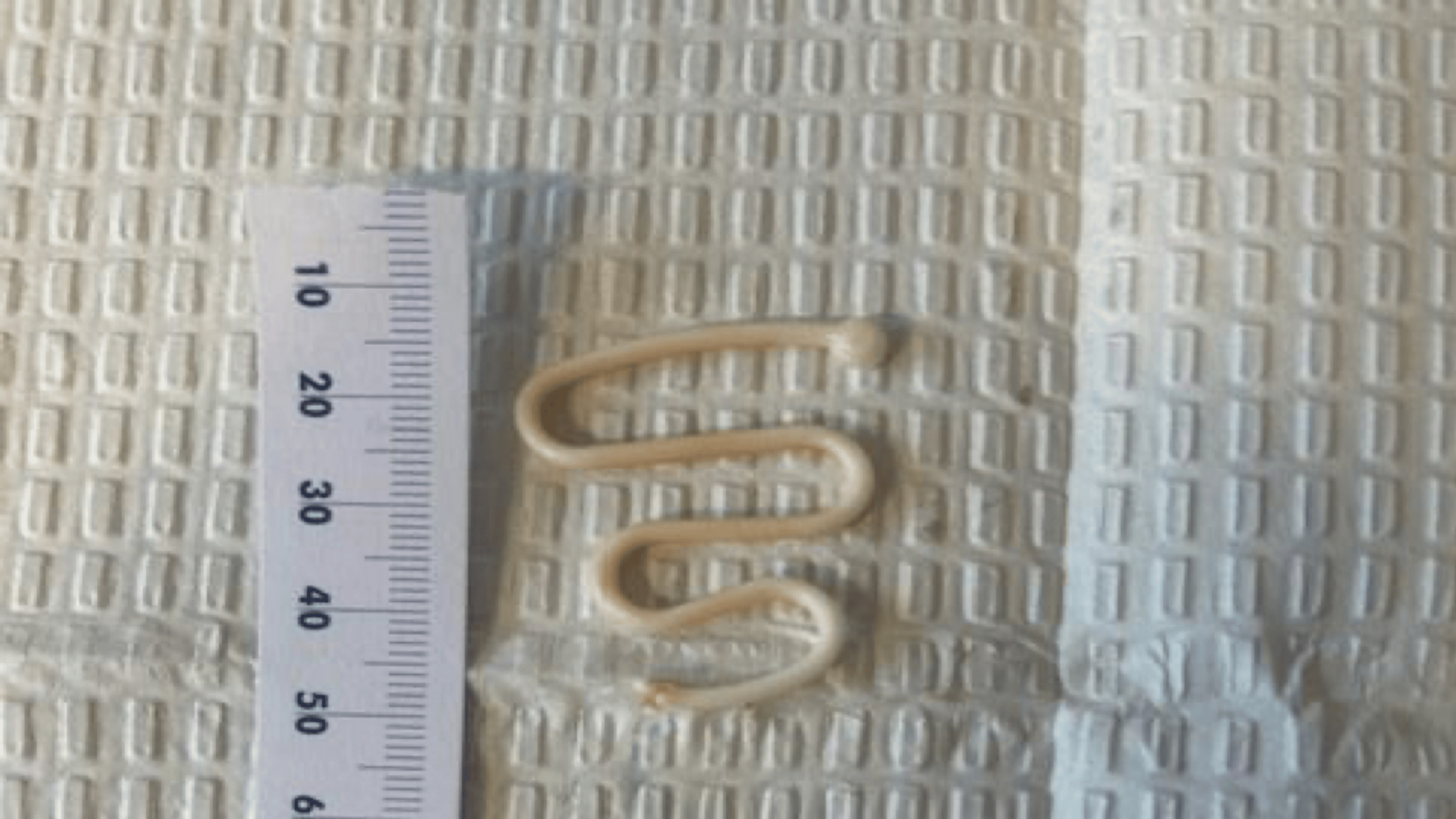
The removed Lippes Loop intrauterine device following laparoscopic extraction

The patient underwent a laparoscopic hysterectomy with bilateral salpingo-oophorectomy due to post-menopausal vaginal bleeding. Intraoperatively, a Lippes Loop IUD was unexpectedly discovered embedded firmly in the cul-de-sac, carefully dissected, and removed laparoscopically without complications (Figure 1). The hysterectomy and salpingo-oophorectomy were completed uneventfully.

Pathological examination of the surgical specimens revealed intramucosal leiomyomas, with no evidence of malignancy. The ovaries and fallopian tubes showed no significant pathology. The foreign body was confirmed to be a Lippes Loop IUD.

The patient tolerated the procedure well, and her postoperative course was uncomplicated. At follow-up visits two and eight weeks postoperatively, her chronic pelvic pain and vaginal bleeding had completely resolved. 

## Discussion

The Lippes Loop intrauterine device, introduced by Dr. Jack Lippes in 1962, represented a significant advancement in contraception during an era of limited options [5]. Constructed from flexible thermoplastic polyethylene with a distinctive trapezoidal double-S design, it was engineered to minimize expulsion. Dr. Lippes also pioneered the use of a monofilament nylon string, greatly simplifying device removal [5]. The Lippes Loop became the most widely used IUD in the United States from the 1960s through the 1980s until its discontinuation by Ortho Pharmaceutical Corporation for economic reasons [6]. Consequently, encountering a Lippes Loop in contemporary practice is exceptionally rare and typically indicates prolonged, often "forgotten," retention. Several case reports have documented long-term retention of Lippes Loop IUDs, with durations ranging from 22 to 44 years [7,8]. The patient was uncertain about the exact date of IUD insertion, so the precise duration of retention could not be determined. Given that Lippes Loop IUDs were manufactured and widely used from 1962 to the 1980s, it is likely that the device had been in situ for decades. This finding is notable, as long-term retention of IUDs, even in asymptomatic patients, can contribute to chronic pelvic pain and emphasizes the importance of considering retained contraceptive devices in the differential diagnosis of postmenopausal pelvic discomfort. Similar to previously described cases, our patient presented with postmenopausal bleeding and chronic pelvic pain [7,8]. The pain associated with long-retained IUDs may result from gradual embedment into the uterine wall, chronic endometrial inflammation, or uterine contractions attempting to expel the device [8,9]. In postmenopausal women, uterine involution may further contribute to device migration and symptom development [7]. A critical aspect of this case is the diagnostic delay. The patient had sought care at multiple healthcare facilities over two years for chronic pelvic pain without receiving a definitive diagnosis. This highlights a recurring challenge in clinical practice: the failure to elicit a complete contraceptive history, particularly in older women whose IUD use may date back decades. Suboptimal counseling at the time of insertion and inadequate documentation have been identified as key factors contributing to prolonged IUD retention [10-12]. When patients present with pelvic pain and a history of IUD use, evaluation should begin with speculum examination to check for IUD strings and transvaginal ultrasound to assess device location [9]. If the IUD is not visualized within the uterus, an abdominal or pelvic X-ray is the next appropriate step to confirm the presence of an extrauterine device [9].

In our case, ultrasound did not demonstrate the IUD, and the patient declined recommended CT imaging, delaying diagnosis until surgery. Uterine perforation by an IUD occurs in approximately 1 in 1,000 insertions, with 80% of perforated devices found within the peritoneal cavity [3,4]. Perforation may be primary, occurring at the time of insertion and often presenting with acute pain, or secondary, resulting from gradual erosion through the myometrium due to chronic pressure and inflammation. In our patient, a history of three cesarean sections may have created areas of myometrial weakness, potentially predisposing her to secondary perforation over decades of device retention. Once extrauterine, IUDs can migrate to various locations, including the cul-de-sac (as in this case), omentum, bowel serosa, bladder, or adjacent viscera [13,14]. While most patients with perforation are asymptomatic at diagnosis [15,16], symptoms such as pain or bleeding may develop over time due to inflammatory adhesions or organ involvement. The management of migrated IUDs warrants careful consideration. Surgical removal is generally recommended, even in asymptomatic patients, to prevent potential complications including bowel or bladder perforation, adhesion formation, infection, and infertility [17,18]. However, the decision must be individualized, weighing the risks of surgery against the likelihood of future complications. In elderly patients with significant comorbidities, expectant management with periodic surveillance may be reasonable for asymptomatic, incidentally discovered migrated IUDs. In our case, given the patient's symptomatic presentation and planned hysterectomy for postmenopausal bleeding, laparoscopic removal was clearly indicated and successfully performed. Minimally invasive approaches, including laparoscopy, are preferred when feasible and when the device is not embedded within viscera [17,18].

This case has several limitations. The exact date and circumstances of IUD insertion could not be ascertained, limiting our ability to determine the precise mechanism and timing of perforation. Additionally, the patient declined cross-sectional imaging, which might have localized the device preoperatively and altered surgical planning. Despite these limitations, this case provides valuable clinical lessons. First, it underscores the importance of a thorough contraceptive history in all women presenting with unexplained pelvic pain, regardless of age. Second, it highlights the need for clear patient counseling at the time of IUD insertion regarding duration of use, recommended timing of removal, and the importance of regular follow-up. Third, it demonstrates that laparoscopic removal of a long-retained, migrated IUD is feasible and effective in symptomatic patients. 

## Conclusions

This case of a 45-year-retained Lippes Loop IUD discovered in a postmenopausal woman with chronic pelvic pain and bleeding illustrates the potential complications of long-term device retention and highlights several important clinical principles. First, we strongly recommend the removal of forgotten IUDs, particularly in postmenopausal women, to prevent complications such as infection, uterine perforation, bleeding, and chronic pelvic pain. Second, patients presenting with postmenopausal bleeding or pelvic pain in the setting of an IUD require thorough evaluation, including careful pelvic examination and appropriate imaging when indicated. Third, this case underscores the critical importance of adequate patient counseling at the time of IUD insertion regarding expected duration of use, potential risks of prolonged retention, and the necessity of regular follow-up and timely removal once the device has expired or is no longer clinically indicated. Ultimately, this case serves as a reminder that even historical devices like the Lippes Loop can have contemporary clinical consequences. Vigilance in contraceptive history-taking, coupled with systematic follow-up and timely intervention, remains essential to preventing avoidable morbidity associated with long-term IUD retention.
